# Comparison of EKFC, Pakistani CKD-EPI and 2021 Race-Free CKD-EPI creatinine equations in South Asian CKD population: A study from Pakistani CKD community cohort

**DOI:** 10.1371/journal.pone.0300428

**Published:** 2024-03-21

**Authors:** Aqsa Safdar, Waqas Akram, Mahtab Ahmad Khan, Danish Tahir, Muhammad Hammad Butt

**Affiliations:** 1 Faculty of Pharmaceutical Sciences, University of Central Punjab, Lahore, Punjab, Pakistan; 2 Department of Medicinal Chemistry, Faculty of Pharmacy, Uppsala University, Uppsala, Sweden; Boston University Chobanian & Avedisian School of Medicine, UNITED STATES

## Abstract

**Introduction:**

South Asian individuals possess a high risk of chronic kidney disease. There is a need to study, evaluate, and compare the newly suggested glomerular filtration rate (eGFR) equations for accurate CKD diagnosis, staging, and drug dosing. This study aimed to (1) evaluate the European Kidney Function Consortium (EKFC), Pakistani CKD-EPI_,_ and 2021 Race-Free CKD-EPI creatinine equation in the South Asian population with CKD and (2) to examine the expected implications on both CKD classification as well as End Stage Renal Disease (ESRD) prevalence across these equations in South Asian population.

**Methods:**

We carried out a cross-sectional investigation on 385 participants, a CKD cohort ≥ 18 years, at Allama Iqbal Medical College, Jinnah Hospital, Lahore. Serum creatinine was measured by Jaffe’s method and rGFR was measured by inulin clearance.

**Results:**

Pakistani CKD-EPI has a lower median difference at -1.33 ml/min/1.73*m*^2^ elevated precision (IQR) at 2.33 (-2.36, -0.03) and higher P30 value at 89.35% than 2021 CKD-EPI and EKFC equations. The mean difference (ml/min/1.73*m*^2^), 95% agreement limits (ml/min/1.73*m*^2^) of CKD-EPI _PK_: -1.18, -6.14, 2021 CKD-EPI: -5.98, -13.24 and EKFC: -5.62, -13.01 (P <0.001). These equations highly correlated to rGFR (P <0.001). An upward re-classification in GFR categories was shown by 2021 CKD-EPI and EKFC compared to the Pakistani CKD-EPI equation. However, there was an exception regarding the G5 category, where an elevated count of 217 (56.36%) was shown for CKD-EPI _PK_. The prevalence of ESRD was seen in entire age groups and prevailed among females more than in males overall equations.

**Conclusions:**

Pakistani CKD-EPI exhibited outstanding performance, while 2021 CKD-EPI and EKFC demonstrated poor performances and could not show an adequate advantage for both CKD classification and prevalence of ESRD compared to Pakistani CKD-EPI. Therefore, Pakistani CKD-EPI appears optimal for this region and warrants future validation in other South Asian countries. In contrast, suitable measures must be implemented in Pakistani laboratories.

## Introduction

According to the 2019 Global Burden of Disease Study, chronic kidney disease (CKD) cases were estimated to be 697.3 million, whereas incident cases were approximately 19 million in 2019 [1]. CKD prevails in around 8.6% of the population all across the world [1]. In the South Asian region, CKD ranges from 10.6% to 23.3% [2], whereas among the Pakistani population, it was estimated to be at 21.2% [2]. Additional studies determined the highest limit of prevalence to be at 29.9% [3] and the lowest at 12.5% [4]. The most beneficial index for the evaluation of kidney function and CKD diagnosis and staging appears to be the glomerular filtration rate (GFR) according to Kidney Disease Improving Global Outcomes (KDIGO) recommendations [5]. In clinical practice, it is unsuitable to directly measure GFR by employing radioisotope or inulin clearance. As a result, endogenous filtration markers are usually used, including serum cystatin C (ScysC) and serum creatinine (SCr) [6].

A novel full-age spectrum eGFR formula was announced by the European Kidney Function Consortium (EKFC), which can be employed over a wide age range [7]. This formula was derived and validated among US and European individuals [8–12], whereas in Asian populations, only a few studies have evaluated it [13, 14]. In 2021, the Chronic Kidney Disease Epidemiology Collaboration (CKD-EPI) recommended novel race-free equations (2021 Race-Free CKD-EPI _Cr_ and CKD-EPI _Cr-CysC_). These formulas were also derived and validated among European and US cohorts [15–18] and comprised very few Asians [15]: One study has evaluated these equations in the South Asia [19].

The current formulas for estimating GFR were evaluated in an innovative and prominent study performed in Karachi, Pakistan, where they were altered and developed in a novel equation that can be implemented in the South Asian region. The CKD-EPI equation was modified using algorithms for intercept and slope, which led to the CKD-EPI Pakistan equation (CKD-EPI_PK_), significantly reducing bias and improving accuracy [20].

Previously, this equation was employed to evaluate the determinants, management and CKD prevalence in the region [4]. This formula has also been used for estimating the changes regarding comorbidities, demographics, and outcomes of CKD [21]. However, limited studies compared it with equations derived for other regions [19, 22]. Moreover, no study has assessed EKFC compared to CKD-EPI_PK_ in South Asia, with only one study evaluating 2021 CKD-EPI compared to the Pakistani CKD-EPI [19]. In this report, we aimed to perform a performance evaluation for 2021 CKD-EPI_,_ Pakistani CKD-EPI, and EKFC equation in South Asian individuals with CKD. Additionally, we aimed to determine the expected implications on the categorization of CKD and the prevalence percentage of End Stage Renal Disease (ESRD) over these equations in the region.

## Methods

### Participant’s characteristics

**Fig 1:**
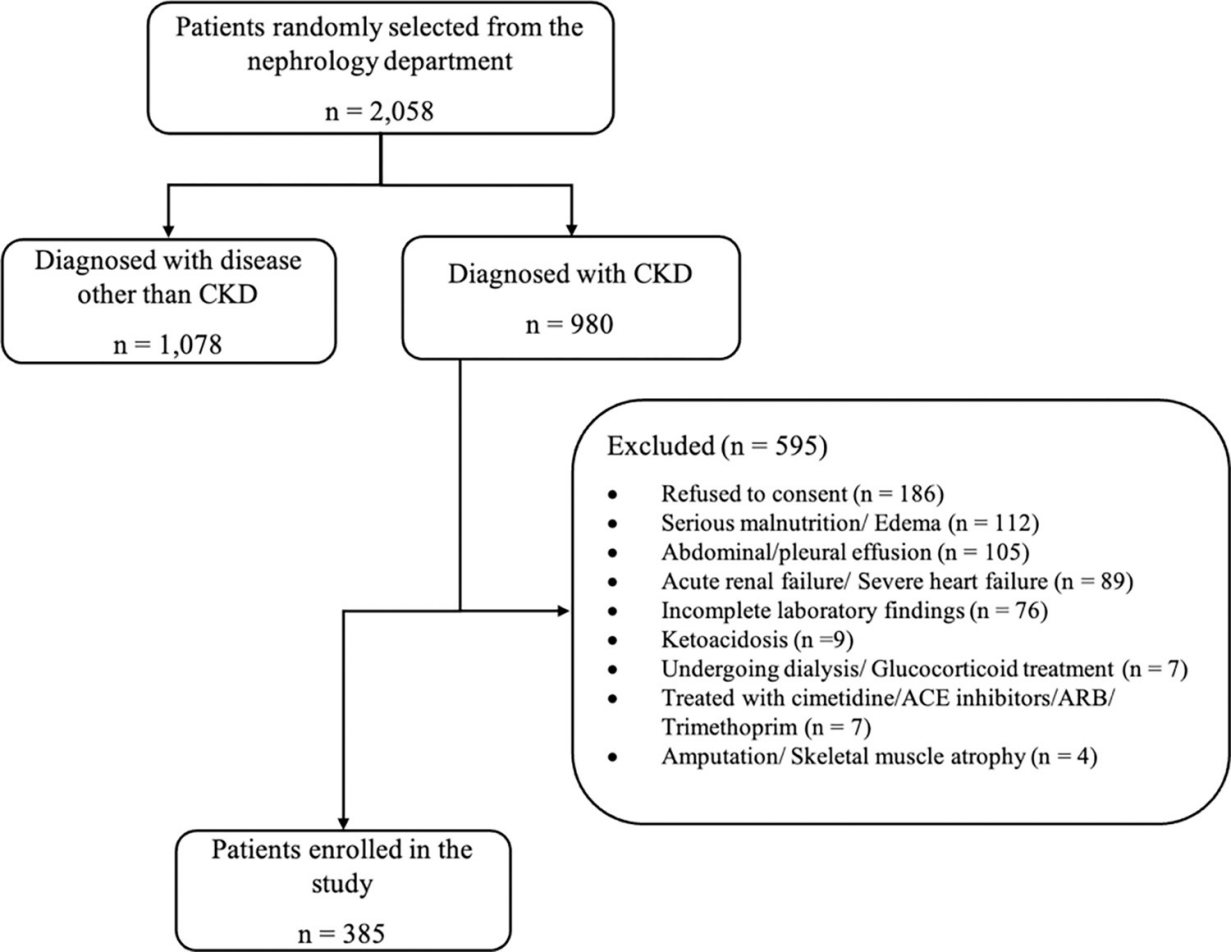
Patient selection flow chart.

We performed a study on a CKD cohort consisting of 385 participants enrolled from 1 November 2021 to 2 March 2023. We employed two novel eGFR equations, 2021 CKD-EPI and EKFC, and a third equation, Pakistani CKD-EPI, on the selected participants. The CKD diagnosis was made according to the criterion of KIDOQI practice guidelines. CKD was diagnosed according to the standard of KIDOQI practice guidelines. The exclusion criteria and some of the study (demographics) have been reported in our previously published paper [23]. Fig 1 shows a patient selection flowchart to clarify the number of patients with each exclusion criterion. Written informed consent was obtained from all study participants. The study was approved by the Ethical Review Board of Allama Iqbal Medical College, Jinnah Hospital, in its 108^th^ meeting held on 23/12/2021 (ERB No. 167/23/12/2021/S2 ERB).

### Measurement of reference GFR (rGFR)

This study used urinary inulin clearance to measure reference GFR (rGFR). Serum and urine concentrations and flow rates were used to estimate inulin clearance. All patients were given a 1% continuous infusion of inulin intravenously for 2 ½ hours after overnight fasting for 12 hours. Hydration among patients was ensured by giving 65 ml water at 29, 59, 89, and 119 minutes. During inulin infusion, serum samples were taken four times (0, 39, 69, and 99 minutes). Urine samples were collected three times (29–59, 59–89, and 89–119 minutes) following the entire bladder emptying at 30 minutes from the time infusion of inulin was started. A kit for the enzymatic method was utilized to assess inulin samples. The three measures were then expressed as a mean value, which acted as rGFR, the gold standard used to compare equations for GFR estimation.

### GFR estimation

GFR estimations were performed with 2021 CKD-EPI, EKFC, and CKD-EPI _PK_ equations. Table 1 enlists the equations studied in the present study.

**Table 1:** Equations included in the study.

No.	Equations	
1.	CKD-EPI _PK_	eGFR = 0.0686×*CKD*−*EPI*^1.059^
3.	EKFC	For age 2–40 years:
		If SCr/Q <1, eGFR = 107.3 × (SCr/Q)^−0.322^
		If SCr/Q ≥1, eGFR = 107.3 × (SCr/Q)^−1.322^
		For age >40 years
		If SCr/Q <1, eGFR = 107.3 × (SCr/Q)^−0.322^ × 0.990^(*Age*−40)^
		If SCr/Q ≥1, eGFR = 107.3 × (SCr/Q)^−1.322^ × 0.990^(*Age*−40)^
4.	2021 CKD-EPI	eGFR = GFR = 142 x min(SCr /κ, 1)^α^ x max(SCr /κ, 1)^-1.200^ x 0.9938^Age^ x 1.012 [if female]
		where,
		SCr = mg/dL, K = 0.7 (females) or 0.9 (males),
		α = -0.241 (females) or -0.302 (males),
		min = indicates the minimum of SCr /K or 1 and
		max = indicates the maximum of SCr /K or 1

Abbreviations: SCr, Serum Creatinine; CKD-EPI _PK_, Pakistani modified equation; 2021 CKD-EPI, 2021 Race-Free CKD-EPI Creatinine equation; EKFC, European Kidney Function Consortium equation

### Laboratory methods

Participants’ blood samples were acquired, and serum creatinine was estimated with the rate of Jaffe reaction performed on Siemens analyzer, ADIVA 2120. Participants’ urine samples were gathered so that estimation of albumin to creatinine ratio (ACR) could be done, which was performed on an ACR analyzer and A1Care ™ HbA1c (Precision; Creatinine: 8% CV, Albumin: ≤ 8% CV).

### Calibration for serum creatinine

Serum creatinine was assayed using a rate-Jaffe reaction performed on a Siemens analyzer, ADIVA 2120. The assay’s calibration was done daily using the two-point method. Calibrators provided by the manufacturer were used, which were traceable to isotope dilution mass spectrometry (IDMS) using the National Institute of Standards and Technology (NIST) creatinine standard reference material (SRM 967). The standardization was done by internal procedures for quality control of the system and by involvement in quality assurance validations performed externally by the College of American Pathologists (CAP).

### CKD classification

CKD was classified into (range ml/min/1.73*m*^2^: stage), 45–59: G3a, 30–44: G3b, 15–29: G4, and <15: G5 as per the KDIGO 2012 recommendations [5].

### Statistical analysis

The data was examined by utilizing IBM-SPSS version 26.0. The three equations were evaluated regarding precision, bias, and accuracy per KDOQI recommendations and the percentage of CKD misclassification. The median difference between rGFR and eGFR was considered as bias. An overestimation was shown as a bias with a negative value and vice versa. The interquartile ranges (IQR) (25^th^ percentage; 75^th^ percentage) among eGFR and rGFR were considered precision. P30 accuracy was expressed as participants’ percentage within the range ±30% rGFR. The limits of agreement and mean differences among each equation were shown by constructing Bland-Altman plots. Linear regression method was used to derive regression equations, and scatter plots were also created. The relationship between rGFR and estimating equations was described by calculating Pearson’s correlation coefficients (r). The coefficient for correlation is categorized as negligible (≤ 0.30), low (0.30–0.49), moderate (0.50–0.69), high (0.70–0.89), and very high (≥ 0.90) [24]. Rates of categorical agreement were calculated when eGFR and rGFR fell within identical GFR categories. The degree of categorical agreements was calculated with weighted kappa value (κ), which is classified as poor (<0.20), fair (0.21–0.40), moderate (0.41–0.60), good (0.61–0.80), and good (>0.81) [25]. ESRD prevalence was expressed as counts and percentages. A P-value of less than 0.001 was considered statistically significant.

## Results

### Baseline demographical data

All of the baseline demographics are represented in Table 2. There were 385 patients, of which 201 (52.2%) were females and 184 (47.79%) were males. Mean ± SD age (years), weight (kg), height (*cm*^2^), BMI (*Kg*/*m*^2^), and BSA (*m*^2^) were 61.99 ± 16.66, 80.14 ± 12.98, 168.19 ± 9.53, 28.56 ± 5.47, and 1.89 ± 0.16 respectively. Median rGFR was 11.23 ml/min/1.73*m*^2^ (IQR: 6.79, 21.2). The median eGFR [IQR ml/min/1.73*m*^2^ (25^th^, 75^th^)] for Pakistani CKD-EPI was 13.59 (8.41, 23.99), for 2021 CKD-EPI was 17.8 (11.47, 30.31) and for EKFC was 16.99 (11, 28.04). The causes of CKD have also been mentioned in Table 2. SCr levels and estimated GFRs have also been classified by age in S1 Table.

**Table 2:** Baseline characteristics (n = 385).

Characteristic	Characteristic
**Gender**	**n (%)**
Male	184 (47.79%)
Female	201 (52.2%)
	**Mean ± SD**
**Age (years)**	61.99 ± 16.66
**Height (*cm*** ^**2**^ **)**	168.19 ± 9.53
**Weight (Kg)**	80.14 ± 12.98
**BSA**^**a**^ **(*m***^**2**^**)**	1.89 ± 0.16
**BMI**^**b**^ **(*Kg*/*m***^**2**^**)**	28.56 ± 5.47
**Serum Creatinine (mg/dL)**	3.72 ± 2.03
**ACR (mg/g)**	188.14 ± 81.56
**BUN (mg/dL)**	40.2 ± 13.76
	**Median (IQR: 25**^**th**^ **- 75**^**th**^ **percentile)**
**rGFR** (ml/min/1.73*m*^2^)	11.23 (6.79–21.2)
**eGFR Pakistani CKD-EPI** (ml/min/1.73*m*^2^)	13.59 (8.41–23.99)
**eGFR 2021 CKD-EPI** (ml/min/1.73*m*^2^)	17.8 (11.47–30.31)
**eGFR EKFC** (ml/min/1.73*m*^2^)	16.99 (11–28.04)
**Causes of CKD**	**n (%)**
Chronic Glomerulonephritis	32 (8.31%)
Hypertensive Nephropathy	78 (20.26%)
Diabetic Nephropathy	83 (21.56%)
Unknown	157 (40.78%)
**CKD Stages** ^**c**^	**n (%)**
Stage G3a	2 (0.52)
Stage G3b	47 (12.20)
Stage G4	113 (29.35)
Stage G5	223 (57.92)

***Abbreviations and definitions*:** BMI, Body Mass Index; BSA, Body Surface Area; ACR, Albumin to Creatinine Ratio; BUN, Blood Urea Nitrogen; rGFR, Reference GFR; eGFR, Estimated Glomerular Filtration Rate; CKD-EPI _PK_, Pakistani Modified equation; EKFC, European Kidney Function Consortium equation; 2021 CKD-EPI, 2021 Race-Free CKD-EPI Creatinine equation

^**a**^ Formula for BSA = 0.007184 × W^0.425^ × H^0.725^

^**b**^ Formula for BMI = $\frac{weight\;(kg)}{height{\;(m)}^{2}}$

^**c**^ Chronic Kidney Disease Epidemiology Collaboration estimated GFR based on Pakistani CKD-EPI <60 ml/min/1.73 m^2^ or urine albumin to creatinine ratio ≥30 mg/g for ≥ 3 months.

### Performance of GFR equations

The median differences for EKFC and 2021 CKD-EPI are -4.48 ml/min/1.73*m*^2^ and -5.59 ml/min/1.73*m*^2^ respectively, while Pakistani CKD-EPI has lower median difference at -1.33 ml/min/1.73*m*^2^ elevated precision (IQR) at 2.33 (-2.36, -0.03) and higher P30 value at 89.35% than 2021 CKD-EPI and EKFC equations (Table 3).

**Table 3:** Performance of included equations.

Equation	Bias*	95% agreement limits (ml/min/1.73m^2^)	Accuracy	Precision
	(ml/min/1.73m^2^)		P30 (%) **	IQR (25^th^, 75^th^)
2021 CKD-EPI	-5.59	-12.6 to 0.64	42.34	4.94 (-8.49, -3.55)
EKFC	-4.48	-12.13 to 0.88	53.51	3.39 (-6.41, -3.02)
CKD-EPI-PK	-1.33	-4.25 to 1.89	89.35	2.33 (-2.36, -0.03)

*Bias is represented as the median difference among eGFR and rGFR

**P30% defined as patients’ percentage with eGFR having ±30% rGFR

**Abbreviations:** eGFR, estimated glomerular filtration rate; CKD-EPI _PK_, Pakistani Modified equation; 202; EKFC, European Kidney Function Consortium equation; 2021 CKD-EPI, 2021 Race-Free CKD-EPI Creatinine equation

### Mean differences and 95% agreement limits

**Fig 2:**
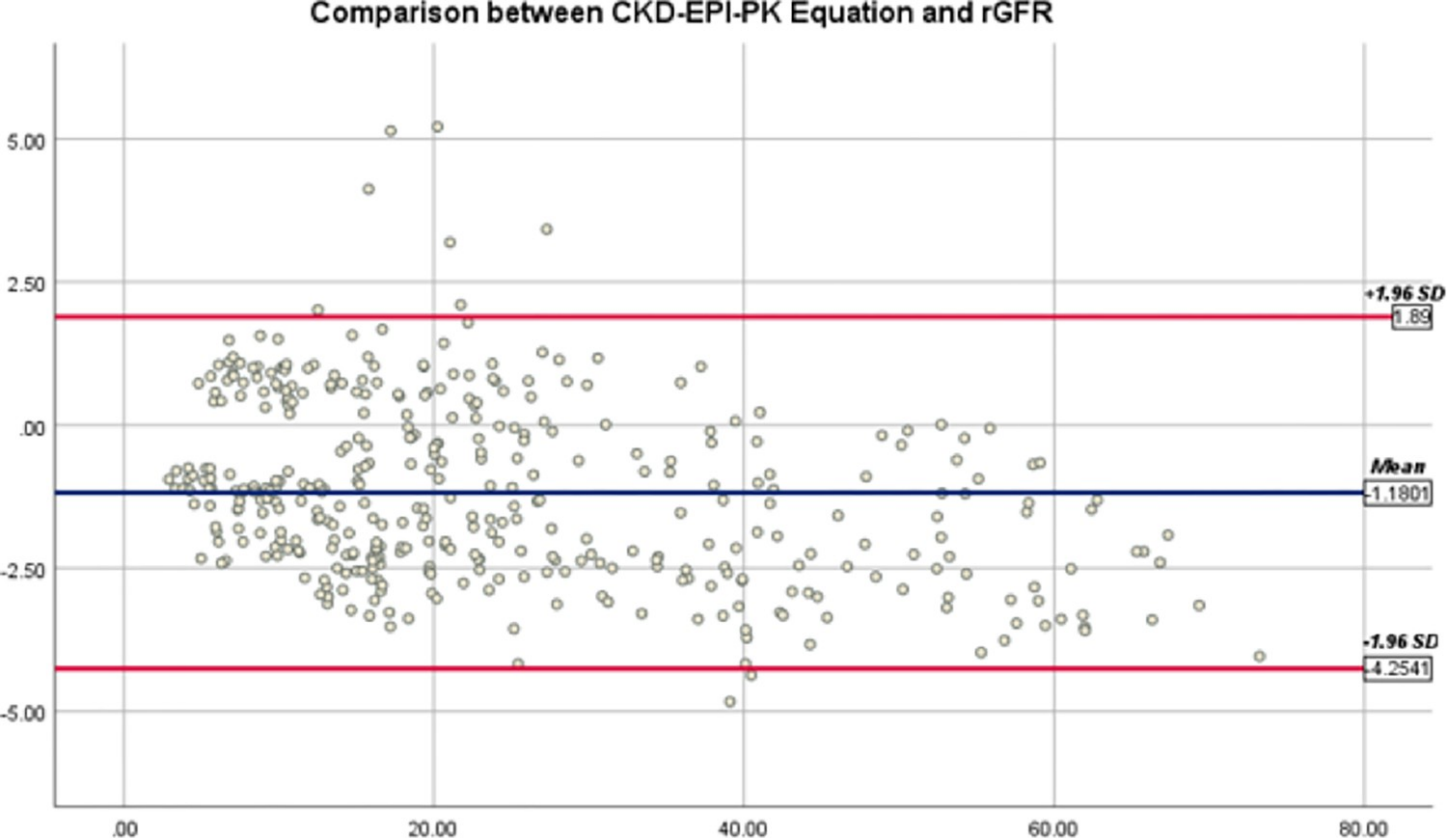
Bland-Altman plot of Pakistani CKD-EPI and rGFR (ml/min/1.73*m*^2^). The mean of Pakistani CKD-EPI plus rGFR is located on the x-axis, and the value of rGFR minus Pakistani CKD-EPI is located on the y-axis. Solid blue line represents mean difference between Pakistani CKD-EPI and rGFR and dark red lines represents 95% limits of agreement of the mean difference between them.

**Fig 3:**
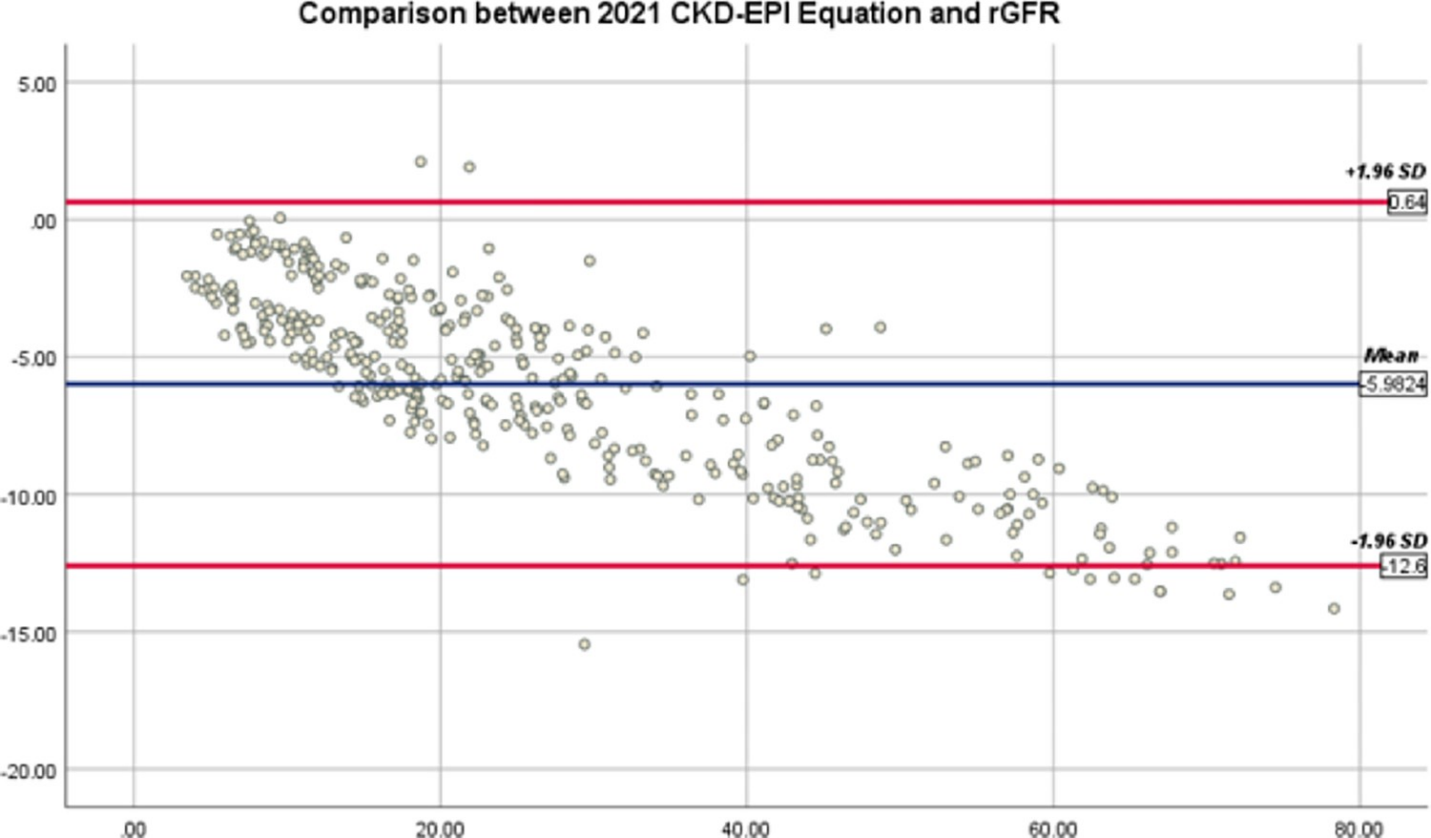
Bland-Altman plot of 2021 CKD-EPI Cr and rGFR (ml/min/1.73*m*^2^). The mean of 2021 CKD-EPI _Cr_ plus rGFR is located on the x-axis, and the value of rGFR minus 2021 CKD-EPI _Cr_ is located on the y-axis. Solid blue line represents mean difference between 2021 CKD-EPI _Cr_ and rGFR and dark red lines represents 95% limits of agreement of the mean difference between them.

**Fig 4:**
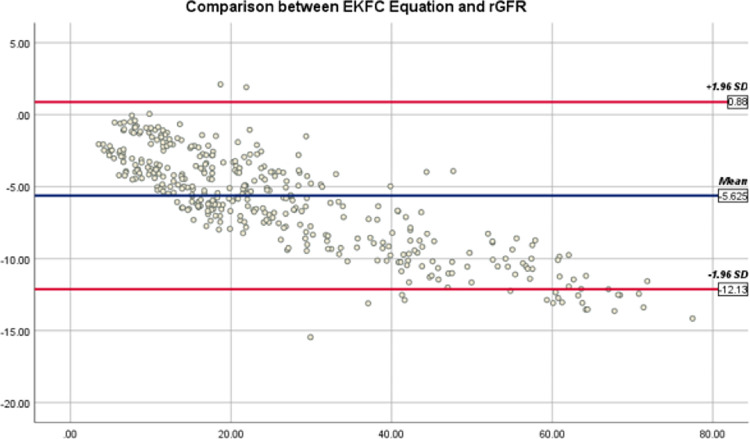
Bland-Altman plot of EKFC and rGFR (ml/min/1.73*m*^2^). The mean of EKFC plus rGFR is located on the x-axis, and the value of rGFR minus EKFC is located on the y-axis. Solid blue line represents mean difference between EKFC and rGFR and dark red lines represents 95% limits of agreement of the mean difference between them.

Bland-Altman graphs demonstrate the mean differences and 95% agreement limits among eGFR by all equations and rGFR (Figs 2–4). The 95% agreement limits (ml/min/1.73*m*^2^) for EKFC and 2021 CKD-EPI equations are broader (−11.25 and −13.24) than Pakistani CKD-EPI equations (-6.14). Elevated negative mean differences for EKFC and 2021 CKD-EPI equations (−5.62 and −5.98) demonstrate that these equations overestimate rGFR in current CKD cohort than Pakistani CKD-EPI equation.

### Regression and correlation analysis

**Fig 5:**
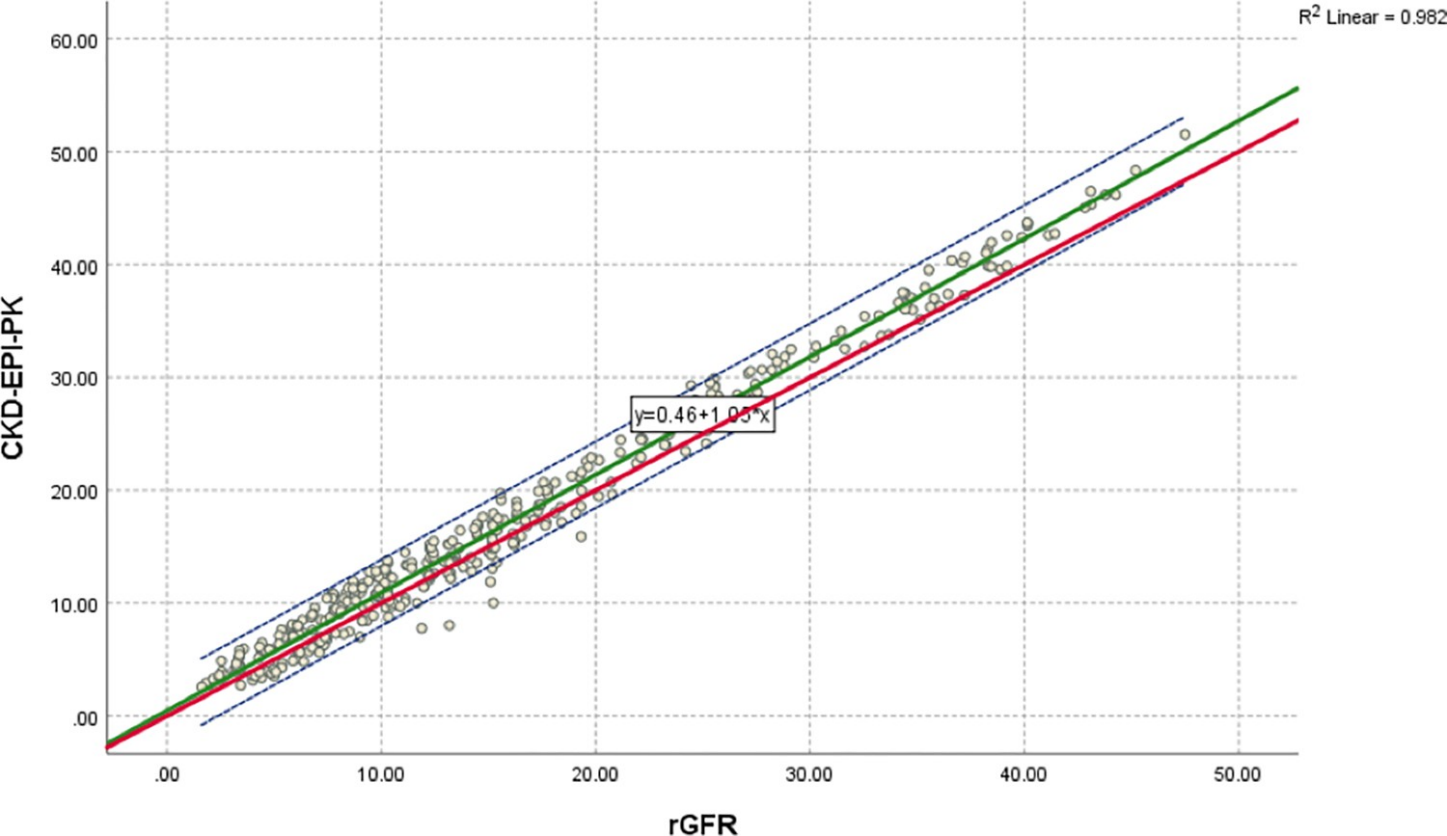
Regression equation and scatter plot of Pakistani CKD-EPI and rGFR (ml/min/1.73*m*^2^). rGFR is located on x-axis, and Pakistani CKD-EPI equation is situated on y-axis. Green line represents the regression line of Pakistani CKD-EPI against rGFR, dashed blue lines shows 95% CI for the regression line. Red line represents the identity line for y = x, respectively. The regression equation of ${eGFR}_{Pakistani\;CKD-EPI}=0.46+1.05*rGFR$, the intercept is below one and the slope is tapered. This scatter diagram represents excellent linear correlation between Pakistani CKD-EPI equation and rGFR, with correlation coefficient of r = 0.982 (P < 0.001). The identity line shows that the Pakistani CKD-EPI equation overestimates eGFR in population of ≤ 60 ml/min/1.73*m*^2^ compared to rGFR.

**Fig 6:**
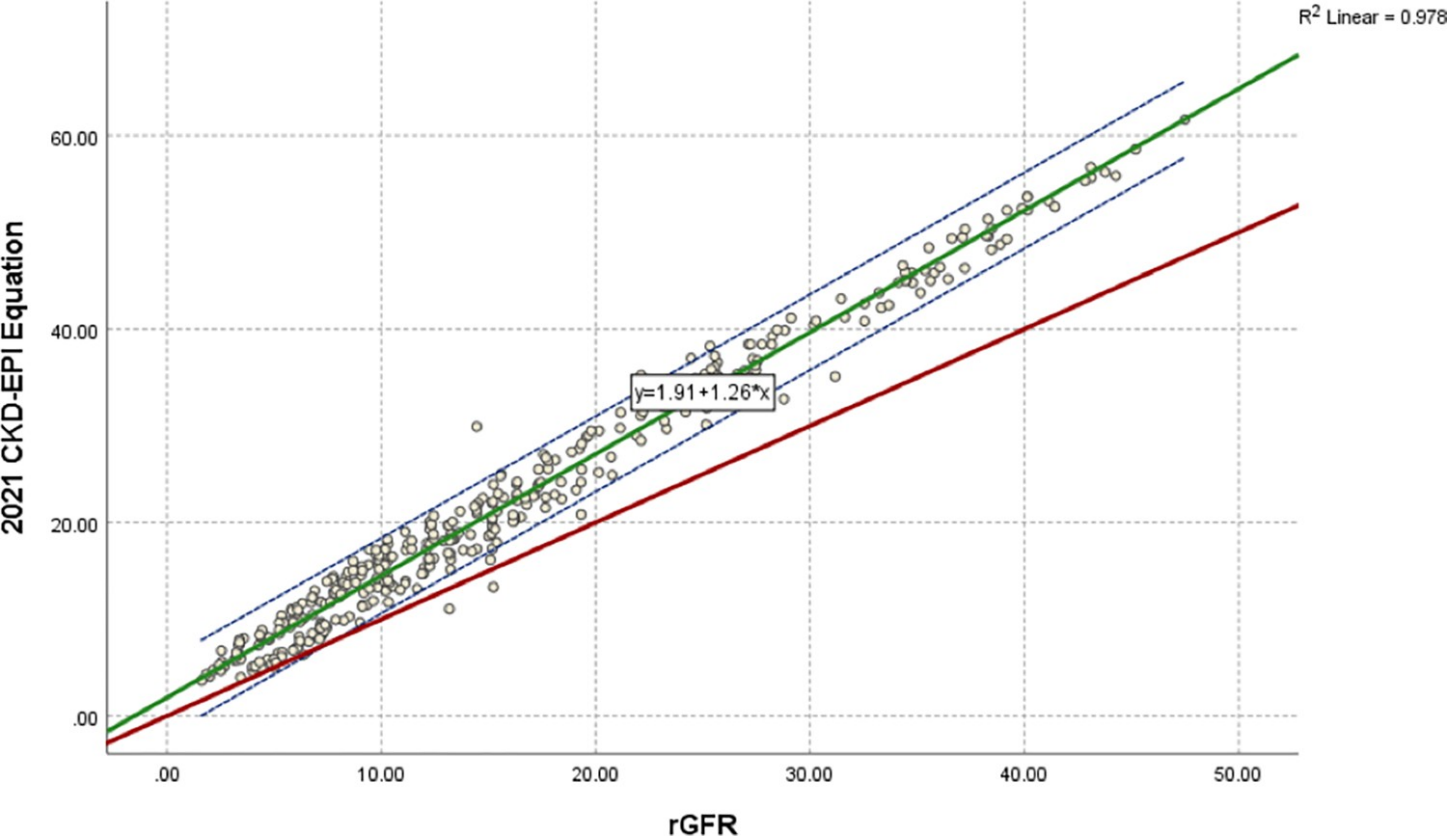
Regression equation and scatter plot of 2021 CKD-EPI and rGFR (ml/min/1.73*m*^2^). rGFR is located on x-axis, and 2021 CKD-EPI equation is situated on y-axis. Green line represents the regression line of 2021 CKD-EPI against rGFR, dashed blue lines shows 95% CI for the regression line. Red line represents the identity line for y = x, respectively. The regression equation of ${eGFR}_{2021\;CKD-EPI}=1.91+1.26*rGFR$, the intercept is nearly two and the slope is tapered. This scatter diagram represents excellent linear correlation between 2021 CKD-EPI equation and rGFR, with correlation coefficient of r = 0.978 (P < 0.001). The identity line shows that 2021 CKD-EPI equation overestimates eGFR in population of ≤ 60 ml/min/1.73*m*^2^ compared to rGFR.

**Fig 7:**
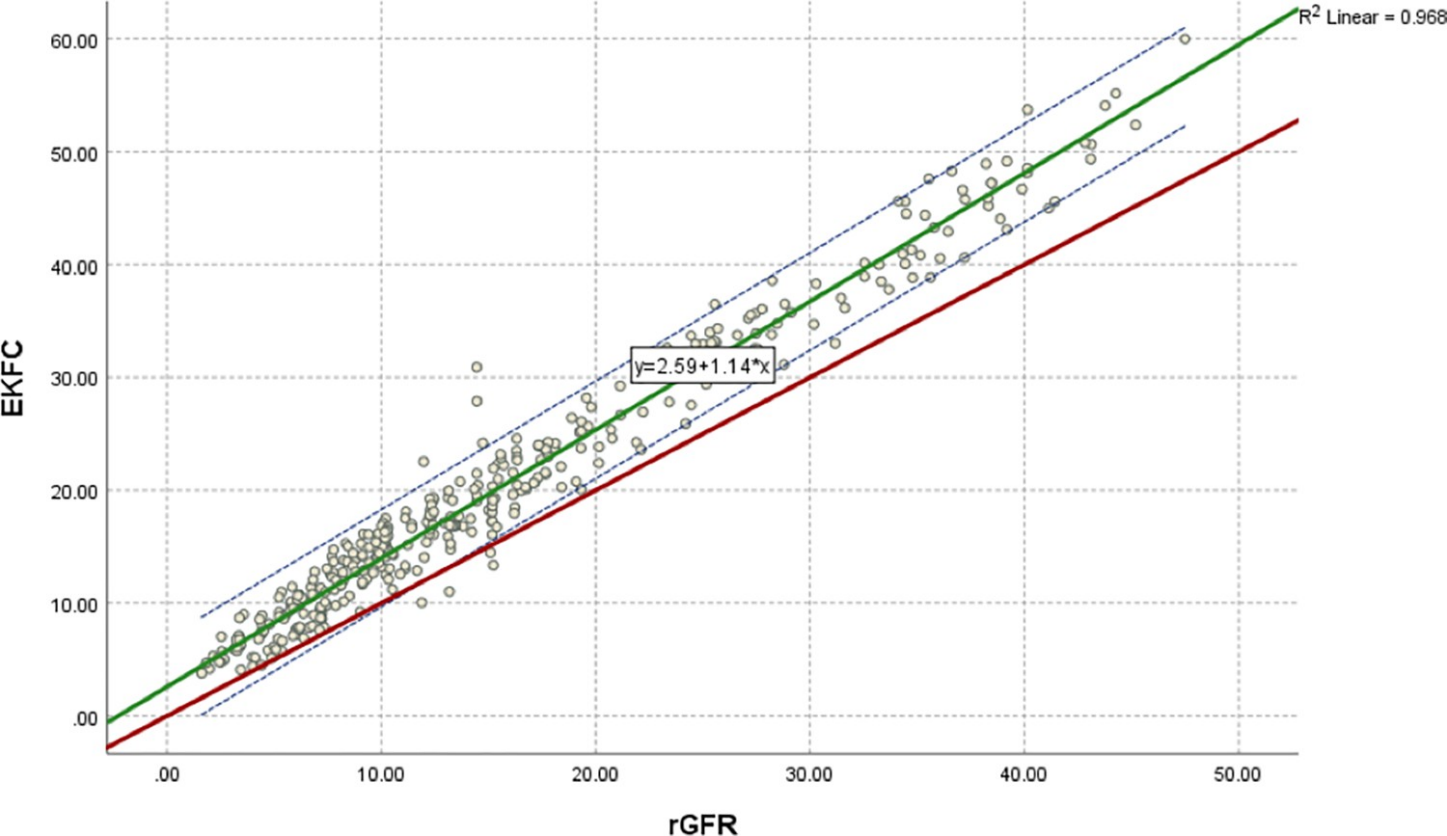
Regression equation and scatter plot of EKFC and rGFR (ml/min/1.73*m*^2^). rGFR is located on x-axis, and EKFC equation is situated on y-axis. Green line represents the regression line of EKFC against rGFR, dashed blue lines shows 95% CI for the regression line. Red line represents the identity line for y = x, respectively. The regression equation of *eGFR*_*EKFC*_ = 2.59+1.14*rGFR, the intercept is nearly three but the slope is tapered. This scatter diagram represents excellent linear correlation between EKFC equation and rGFR, with correlation coefficient of r = 0.968 (P < 0.001). The identity line shows that EKFC equation overestimates eGFR in population of ≤ 60 ml/min/1.73*m*^2^ compared to rGFR.

Fig 5 indicates a regression equation and a scatter plot for rGFR and CKD-EPI _PK_. The regression equation of ${eGFR}_{CKD-EPI-PK}=0.46+1.05*rGFR$, the slope is under one, and the intercept has tapered. Fig 6 indicates a regression equation and a scatter plot for the rGFR and 2021 CKD-EPI equation. The regression equation of ${eGFR}_{2021\;CKD-EPICr}=1.91+1.26*rGFR,$ the slope is nearly two, and the intercept has also tapered. Fig 7 indicates a regression equation and a scatter plot for rGFR and EKFC. The regression equation of ${eGFR}_{EKFC}=2.59+1.14*rGFR$, the slope is nearly three, but the intercept has tapered. Conforming with Pearson’s correlation (r) and linear regression, Pakistani CKD-EPI matches rGFR closely compared to the 2021 CKD-EPI and EKFC equation. The coefficients are 0.982, 0.978, and 0.968 for Pakistani CKD-EPI_,_ 2021 CKD-EPI, and EKFC, respectively, compared to rGFR. Therefore, these formulas are highly correlated to rGFR.

### Categorical agreement rates

The categorical agreement rates are 90.9%, 65.71%, and 60.26% for CKD-EPI _PK_, EKFC, and 2021 CKD-EPI formulas respectively. Hence, the more robust categorical agreement is depicted by the EKFC equation compared to 2021 CKD-EPI_,_ whereas categorical agreement for Pakistani CKD-EPI was the highest. Upward reclassification (G3b < G3a and G4 < G3b) when using 2021 CKD-EPI and EKFC equations compared to rGFR with an exception for the G5 stage. Discordant KIDGO CKD classification and percentage of categorical agreement among Estimating Equations and rGFR is shown in Table 4. Weighted kappa values are ‘good’ in the case of 2021 CKD-EPI, whereas ‘very good’ in the EKFC and Pakistani CKD-EPI equation.

**Table 4:** Discordant KIDGO CKD classification and percentage of categorical agreement among estimating equations and rGFR.

Equation	GFR Categories ^a^	CKD categorization established on rGFR					Categorical Agreement	
		Stage G3a	Stage G3b	Stage G4	StageG5	Total	%	Weighted Kappa (κ) (95% CI)
CKD-EPI-PK	G3a	**2**	5	0	0	7	90.9	0.955 (0.948–0.962)
	G3b	0	**42**	9	0	51		
	G4	0	0	**97**	13	110		
	G5	0	0	8	**209**	217		
2021 CKD-EPI	G3a	**2**	27	0	0	29	60.26	0.801 (0.789–0.813)
	G3b	0	**20**	48	0	68		
	G4	0	0	**65**	77	142		
	G5	0	0	1	**145**	146		
EKFC	G3a	**2**	21	0	0	23	65.71	0.829 (0.817–0.841)
	G3b	0	**26**	41	1	68		
	G4	0	0	**71**	67	138		
	G5	0	0	2	**154**	156		
Total		2	47	114	222	385		

Abbreviations: rGFR, Reference GFR; CKD-EPI _PK_, CKD-EPI equation with Pakistani Modification Factors; 2021 CKD-EPI, 2021 Race-Free CKD-EPI Creatinine equation; EKFC, European Kidney Function Consortium equation

^a^ Definitions as per KDIGO guidelines

### Prevalence of End Stage Renal Disease (ESRD)

The End Stage Renal Disease (ESRD) (≤15 ml/min/1.73m^2^) prevalence has been classified according to age groups and gender (S2 Table). The Pakistani CKD-EPI formula generally estimated ESRD prevalence at 56.36%, which was also the closest to rGFR, while the 2021 CKD-EPI equation had the lowest estimation at 37.92. The prevalence of ESRD is observed in entire age groups, while it prevails among females more than in males overall equations.

## Discussions

According to the recommendations made by KDIGO, the CKD-EPI equation can only be used if another formula has not been demonstrated to be superior in accuracy and reliability in the region [5]. CKD-EPI equations are used in North America, Europe, and Australia; various eGFR equations, after sufficient evaluation, can be applied to other populations [5, 6, 26]. The current study is the foremost validation study assessing the EKFC equation with reference to measured GFR in the South Asian population. However, 2021 CKD-EPI and Pakistani CKD-EPI have been validated once in a previous study in this population [19]_._ These equations were assessed and compared in a CKD cohort in the South Asian region according to the recommendations made by KDIGO, and their expected implications were also determined in terms of GFR classification and prevalence of ESRD.

In the current study, the best performance [Bias: -1.33 ml/min/1.73*m*^2^, IQR 25^th^, 75^th^: 4.94 (-8.49, -3.55), P30: 89.35%] has been shown by CKD-EPI _PK,_ a formula with Pakistani modified algorithms. Hence, this study’s outcomes confirm the study’s findings for developing this equation [20]. In addition, this equation has demonstrated an ideal performance in moderate to severe CKD. However, the development study needed more individuals with low GFR values and extensive performance evaluation in various CKD stages [20]. A high categorical agreement at 90.9% with rGFR has also been shown by this equation. In addition, the equation was also linked to a high ESRD proportion that was identical to earlier analysis [4, 21].

Pakistani CKD-EPI equation was produced by obtaining the correction factors derived by linear regression representations of natural logarithms of rGFR against natural logarithms of eGFR estimated by the CKD-EPI formula. The slopes and intercepts, which were statistically similar, were then adapted to exponential form. These were used as correction elements to change the formula to Pakistani CKD-EPI [20]_._ This formula is clinically significant as CKD was linked independently with hypertension, diabetes, elderly age, raised systolic BP, elevated triglycerides, elevated glucose levels, and stroke history in this study [27]. Also, it was previously utilized to evaluate the changes in CKD associated with comorbidities, demographics, and outcomes. Each 1mL/min/1.73m2 decrease in Pakistani CKD-EPI was linked with a 13.1% elevated ESRD risk [21, 28]. Regarding cystatin-C-based CKD-EPI, according to a study, 2012 CKD-EPI _CysC_ exhibited substantial bias in the Pakistani population, and 2012 CKD-EPI _Cr-CysC_ was no better than Pakistani CKD-EPI [28].

The other equation close to rGFR after Pakistani CKD-EPI is EKFC (mean difference -4.4; 95% agreement limits -13.01) (Fig 4). This equation has a conceptual advantage as it can be utilized for all ages, and it removes the discontinuity in estimated GFR in transitioning from adolescence to adulthood. In addition, it does not overestimate GFR among young adults. However, our study observed an upward GFR category reclassification trend with EKFC and an overestimated rGFR in the South Asian CKD population. However, the categorical agreement between EKFC and rGFR was 65.71% (Table 4).

However, according to the recent statement of the American Society of Nephrology Task Force and the National Kidney Foundation, the transformers for the ethnicity ought to be excluded in formulas for estimation of kidney function [16]. This study reported higher *eGFR*_2021 *CKD−EPI*_ mean difference and more comprehensive 95% agreement limits (-5.98, -13.24) than the Pakistani CKD-EPI (-1.18, -6.14) (Figs 2 and 3). These results are similar to the previous findings, which suggest that the race-free 2021 eGFR equations are not better than *eGFR*_*CKD*−*EPI*−*PK*_. However, some studies reported that removing the race coefficient from the CKD-EPI equation increases bias instead of improving its accuracy [29], which is similar to our findings. However, other studies do not support the need for consideration of race in the estimation of GFR and additional clinical algorithms [30, 31], but these studies had the limitation that they did not have a robust representation of the Asian population and other ethnic or racial subgroups.

Race is not just Blacks or non-Blacks. Americans and Europeans have a distinct physical composition from Asians [32]; for example, there is a difference in muscle mass and waist circumference. Thus, Asian-specific coefficients must be taken into consideration. According to a recent study in the Chinese population [33], the race-free equations overestimated the reference GFR, and the 2021 CKD-EPI equation was worse than the 2009 CKD-EPI equation regarding P30 and precision in Chinese individuals. Our study depicts similar findings. Hence, it is vital to consider the race coefficients for other populations.

The distribution of CKD categories varied across the equations, particularly for the 2021 CKD-EPI formula. Conforming to CKD-EPI _PK,_ patients’ proportion classified in the G5 stage exceeded 50% and showed closely identical classification to rGFR. This also illuminates the significance of considering race coefficients for various racial groups in 2021 CKD-EPI and EKFC formulas to categorize different CKD categories accurately. In addition, South Asians are already a high-risk population for ESRD, and using these equations would result in the diagnosis and treatment of individuals with high-risk and over diagnosed stages from G3a to G4.

The mean value of rGFR was 15.73ml/min/1.73*m*^2^, which was correlatively lower compared to 2021 CKD-EPI and EKFC equations. Therefore, upward reclassification can easily be noticed in this study. An optimal performance has been shown by CKD-EPI _PK,_ which indicates that GFR values might be less for individuals in this region compared to the populations in Western and other Asian areas with distinct races or ethnicities. This also calls attention to altering the range of CKD classification for this region and re-considering the CKD cut-off value (60 ml/min/1.73m squared) so that individuals can be categorized accurately under different CKD classifications. This outcome is similar to the earlier research [4].

The present study’s ESRD prevalence varied according to the equation (S2 Table). Although ESRD existed in all age groups, higher percentages of the majority were seen in women conforming to all equations. In addition, elevated proportions of ESRD can also be seen in individuals in their 80s for Pakistani CKD-EPI, as well as EKFC equations. The findings were also similar to earlier studies [27, 34]. Gender ratios, KDIGO risk categorization, and CKD prevalence changed dramatically in Southeast Asia based on the estimating formula used by [35].

This study has several strengths. Firstly, urinary inulin clearance has been employed as a reference for accurate GFR measurement. Secondly, this study is the first validation analysis performed externally for the EKFC equation in a CKD cohort in the South Asian region against inulin clearance. Thirdly, we enrolled participants from the kidney center of a well-known government hospital with an outstanding turnover rate for renal patients from all cities across Pakistan. Therefore, the results of this study can be considered exclusively, at least for this area. Lastly, we estimated ESRD prevalence based on eGFR and albuminuria, magnifying this study’s robustness [5, 18].

There are certain limitations as well that must be noted. Firstly, the equations considered in the current study possess certain restrictions, To bring out the correct eGFR value, they need to be further optimized and modified [7, 15, 20]. Secondly, we kept out participants younger than 18 years, and more than this, eliminating criterion is needed for inspection of the familiar advantage concerning the EKFC formula, which is appropriate to the entire age spectrum. However, other formulas included in this study were developed in individuals aged ≥ 18 years; hence, disparities would be introduced in results if individuals aged < 18 years were included. Thirdly, recommended procedures were used to estimate serum creatinine levels with adequate standardization and quality control, although analytical and biological differences can inevitably affect these levels. These measurement uncertainties cannot be entirely avoided in the current study [36]. Finally, although the Jaffe method used in our study would have increased the misclassification of CKD into various stages, the potential for misclassification is lower as it was based on not only as long as the eGFR had been low for ≥ 3 months but also on albuminuria.

In conclusion, the best performance has been shown by Pakistani CKD-EPI with high precision, low bias, and P30 accuracy, while on the contrary, poor performances have been demonstrated by 2021 CKD-EPI and EKFC and they had no adequate advantage over Pakistani CKD-EPI in terms of GFR categorization and estimation of ESRD prevalence. Therefore, Pakistani CKD-EPI appears ideal for this region and warrants future validation in other South Asian countries. Besides, suitable measures must also be taken to implement this equation across Pakistani laboratories.

## Supporting information

S1 Checklist STROBE statement—checklist of items that should be included in reports of observational studies. (DOCX)

S1 File Inclusivity in global research. (DOCX)

S1 Table Stratification of estimated glomerular filtration rate and serum creatinine by age groups. (DOCX)

S2 Table Prevalence of End Stage Renal Disease (ESRD) (≤15 ml/min/1.73m^2^) by rGFR and each equation, stratified by age group and gender. (DOCX)

S1 Data (XLSX)
