# Supplementary material for: Comparison of EKFC, Pakistani CKD-EPI and 2021 Race-Free CKD-EPI creatinine equations in South Asian CKD population: A study from Pakistani CKD community cohort
Source: PLoS One. 2024 Mar 21;19(3):e0300428. doi: 10.1371/journal.pone.0300428 (PMC10956795; doi:10.1371/journal.pone.0300428)
Supplement: S1 Table — (DOCX) [file pone.0300428.s003.docx]

**Table S1.** Stratification of Estimated Glomerular Filtration Rate and Serum Creatinine by Age Groups

| **Age Range** | **Serum Creatinine**  **(mg/dL)** | **eGFR Pakistani CKD-EPI**  **(ml/min/1.73**$\boldsymbol{m}^{\boldsymbol{2}}$**)** | **eGFR 2021 CKD-EPI**  **(ml/min/1.73**$\boldsymbol{m}^{\boldsymbol{2}}$**)** | **eGFR EKFC**  **(ml/min/1.73**$\boldsymbol{m}^{\boldsymbol{2}}$**)** |
| --- | --- | --- | --- | --- |
| 20-29 years (N = 16) | 4.19±2.18 | 19.73±13.84 | 24.33±16.25 | 23.46±13.31 |
| 30-39 years (N = 33) | 4.18±1.98 | 17.02±11.83 | 21.71±14.09 | 21.93±13.55 |
| 40-49 years (N = 49) | 3.8±1.8 | 16.88±10.57 | 21.47±12.59 | 21.83±12.2 |
| 50-59 years (N = 54) | 3.8±2.19 | 17.31±11.32 | 22.03±13.67 | 21.4±12.66 |
| 60-69 years (N = 97) | 3.76±2.27 | 17.62±12.47 | 22.49±15.03 | 20.95±13.38 |
| 70-79 years (N =83) | 3.24±1.67 | 17.27±10.46 | 22.44±12.75 | 20.14±10.9 |
| 80-89 years (N = 45) | 3.65±1.92 | 14.03±8.77 | 18.57±10.95 | 16.27±9.08 |
| 90-99 years (N = 8) | 4.1±2.67 | 12.27±10.83 | 16.47±10.49 | 13.67±8.13 |

All data presented are expressed as mean ±standard deviation

Abbreviations: eGFR, Estimated Glomerular Filtration Rate; Pakistani CKD-EPI, CKD-EPI equation with Pakistani Modification Factors; 2021 CKD-EPI, 2021 Race-Free CKD-EPI creatinine equation; EKFC, European Kidney Function Consortium equation;
