# Supplementary material for: Comparison of EKFC, Pakistani CKD-EPI and 2021 Race-Free CKD-EPI creatinine equations in South Asian CKD population: A study from Pakistani CKD community cohort
Source: PLoS One. 2024 Mar 21;19(3):e0300428. doi: 10.1371/journal.pone.0300428 (PMC10956795; doi:10.1371/journal.pone.0300428)
Supplement: S2 Table — (DOCX) [file pone.0300428.s004.docx]

**Table S2.** Prevalence of End Stage Renal Disease (ESRD) ( ≤15 ml/min/1.73$m^{2}$) by rGFR and Each Equation, Stratified by Age Group and Gender

|  | **Number (%) of patients with End-Stage Renal Disease by each equation** | | | | | | | | | | | |
| --- | --- | --- | --- | --- | --- | --- | --- | --- | --- | --- | --- | --- |
|  | **Total (n=385)** | | | | **Male (n=184)** | | | | **Female (n=201)** | | | |
| **Age Group (years)** | rGFR | Pakistani CKD-EPI | 2021 CKD-EPI | EKFC | rGFR | Pakistani CKD-EPI | 2021 CKD-EPI | EKFC | rGFR | Pakistani CKD-EPI | 2021 CKD-EPI | EKFC |
| 20-29 | 9 (56.25) | 9 (56.25) | 8 (50.00) | 7 (43.75) | 3 (37.5) | 3 (37.5) | 3 (37.5) | 3 (37.5) | 6 (75.00) | 6 (75.00) | 5 (62.5) | 4 (50.00) |
| 30-39 | 22 (66.66) | 21 (63.63) | 12 (36.36) | 10 (30.30) | 11 (57.89) | 10 (52.63) | 6 (31.58) | 4 (21.05) | 11 (78.57) | 11 (78.57) | 6 (42.86) | 6 (42.86) |
| 40-49 | 31 (63.27) | 28 (57.14) | 19 (38.78) | 17 (34.69) | 14 (100.00) | 12 (50.00) | 7 (29.17) | 5 (20.83) | 17 (100.00) | 16 (64.00) | 12 (48.00) | 12 (48.00) |
| 50-59 | 26 (48.15) | 26 (48.15) | 21 (38.89) | 22 (40.74) | 6 (27.27) | 6 (27.27) | 4 (18.18) | 5 (22.73) | 20 (62.5) | 20 (62.5) | 17 (53.12) | 17 (53.13) |
| 60-69 | 57 (58.76) | 53 (54.63) | 38 (39.18) | 40 (41.24) | 22 (46.80) | 20 (42.55) | 14 (29.79) | 14 (29.79) | 35 (70.00) | 33 (66.00) | 24 (48.00) | 26 (52.00) |
| 70-79 | 43 (51.80) | 45 (54.22) | 25 (30.12) | 27 (32.53) | 19 (50.00) | 19 (50.00) | 7 (18.42) | 9 (23.68) | 24 (53.33) | 26 (57.78) | 18 (40.00) | 18 (40.00) |
| 80-89 | 29 (64.44) | 29 (64.44) | 19 (42.22) | 27 (60.00) | 13 (59.00) | 13 (59.09) | 9 (40.9) | 12 (54.55) | 16 (69.57) | 16 (69.57) | 10 (43.48) | 15 (65.22) |
| 90-99 | 6 (75.00) | 6 (75.00) | 4 (50.00) | 6 (75.00) | 4 (100.00) | 4 (100.00) | 4 (100.00) | 4 (100.0) | 2 (50.00) | 2 (50.00) | 0 (00.00) | 2 (50.00) |
| Total | 9 (56.25) | 217 (56.36) | 146 (37.92) | 156 (40.52) | 92 (50.00) | 87 (47.28) | 54 (29.35) | 56 (30.43) | 131 (65.17) | 130 (64.68) | 92 (45.77) | 100 (49.75) |

Abbreviations: rGFR, Reference GFR; Pakistani CKD-EPI, CKD-EPI equation with Pakistani Modification Factors; 2021 CKD-EPI, 2021 Race-Free CKD-EPI Creatinine equation; EKFC, European Kidney Function Consortium equation

Note: Percentage (%) of ESRD prevalence in each age group is calculated by the number of individuals with ESRD (≤15 ml/min/1.73$m^{2}$) divided by the total number of individuals in that age group.
